# Complete Genome Sequence of the Hyperthermophilic Piezophilic Archaeon *Pyrococcus kukulkanii* NCB100 Isolated from the Rebecca’s Roost Hydrothermal Vent in the Guaymas Basin

**DOI:** 10.1128/genomeA.01667-16

**Published:** 2017-02-16

**Authors:** Philippe M. Oger, Nolwenn Callac, Christine Oger-Desfeux, Sandrine Hughes, Benjamin Gillet, Mohamed Jebbar, Anne Godfroy

**Affiliations:** a CNRS, UMR 5276, Laboratoire de Géologie de Lyon, Lyon, France; b Université de Brest, UEB, IUEM, Laboratoire de Microbiologie des Environnements Extrêmes, UMR 6197, Plouzané, France; c Ifremer, Laboratoire de Microbiologie des Environnements Extrêmes, UMR 6197, Technopôle Brest Iroise, Plouzané, France; d CNRS, Laboratoire de Microbiologie des Environnements Extrêmes, UMR 6197, Plouzané, France; e Université de Brest, Domaines Océaniques IUEM, UMR 6538, Plouzané, France; f Pôle Rhône Alpes de Bioinformatique, Université Claude Bernard Lyon 1, Villeurbanne, France; g École Normale Supériure de Lyon, Institut de Génomique Fonctionnelle de Lyon, Lyon, France

## Abstract

Members of the order *Thermococcales* are common inhabitants of high-temperature hydrothermal vent systems (black smokers) that are represented in clone libraries mostly by isolates from the *Thermococcus* genus. We report the complete sequence of a novel species from the *Pyrococcus* genus, *P. kukulkanii* strain NCB100, which has been isolated from a flange fragment of the Rebecca’s Roost hydrothermal vent system in the Guaymas Basin.

## GENOME ANNOUNCEMENT

*Pyrococcus kukulkanii* strain NCB100^T^ was isolated from a flange fragment collected at the Rebecca’s Roost hydrothermal vent in the Guaymas Basin at a depth of 2004 m by cultivation in SME medium at 100°C (1). *P. kukulkanii* is the most hyperthermophilic *Thermococcales* known to date, with an optimum growth temperature of 105°C. This species is a piezophilic, obligately anaerobic chemoorganotroph, growing best by fermentation of proteinaceaous substrates, such as peptone, in the presence of sulfur.

DNA was extracted from cells grown in SME medium to late exponential phase as described in Barbier et al (2). For determination of the complete genome sequence, a first 800 bp fragment library was sequenced with Ion Torrent PGM using a 316 chip and the HiQ, which generated 583 k reads with a mean 389 bp length after QC check and trimming (http://www.bioinformatics.babraham.ac.uk/projects/) (3). A second 500 bp paired-end library was sequenced (2 × 100 bp) as part of a HiSeq run, which generated 13 million paired 100 bp reads after QC check and trimming (3). The IonTorrent data were assembled *de novo* with Newbler 2.8. The paired-end Illumina data was assembled *de novo* with velvet (4). The pooled IonTorrent and Illumina data was assembled *de novo* with MIRA (5) and Newbler. The resulting assemblies generated 27, 11, 2, and 2 contigs for IonTorrent, Illumina, and both hybrid assemblies, respectively. The scaffolds were connected by Sanger sequencing of PCR amplified fragments. The total size of the assembly is a single circular chromosome of 1,977,126 bp with an average G+C content of 44.6%. The sequence was automatically annotated by the NCBI (6) and is available on the MaGe platform for genomic comparisons (7, 8).

The genome consists of 2,207 protein-coding genes, 47 tRNAs, two copies of the 5S rRNA, and one copy of the 16S-23S genes. With inclusion of strain NCB100, the new core- and pan-genome of the *Pyrococcus* genome contains 1,086 and 4,539 genes families, respectively. Most (393/427) of the *P. kukulkanii* specific genome comprises open reading frames (ORFs) with no known functions. Strain NCB100 is most closely related to *Pyrococcus* sp. strain ST04, which has been isolated from the Juan de Fuca Ridge (9). *In silico* hybridization yielded an average nucleotide identity (ANI) value (of protein-coding genes shared at ≥60% nucleotide identity and ≥70% coverage) of 78%, over 1,318 genes conserved between the two species, which indicate phylogenetically close but distinct species (10). Genomic comparisons reveal an organotrophic potential comparable in both species (6, 7). In contrast to other *Pyrococcus* species, *P. kukulkanii* encodes the locus for formate-driven hydrogenogenesis growth described so far only in *Thermococcus onnurineus*, *T. gammatoelrans*, and *T. barophilus* (11–13) and present only in the other piezophile *Pyrococcus yayanosii* (14). However, it lacks the CO dehydrogenase which allows hydrogenogenic lithoheterotrophic growth on CO as in these three *Thermococcus* species (15).

### Accession number(s).

The final annotated genome of *Pyrococcus kukulkanii* NCB100 is available in GenBank under accession number CP010835.
